# *Candida albicans OPI1* Regulates Filamentous Growth and Virulence in Vaginal Infections, but Not Inositol Biosynthesis

**DOI:** 10.1371/journal.pone.0116974

**Published:** 2015-01-20

**Authors:** Ying-Lien Chen, Flavia de Bernardis, Shang-Jie Yu, Silvia Sandini, Sarah Kauffman, Robert N. Tams, Emily Bethea, Todd B. Reynolds

**Affiliations:** 1 Department of Plant Pathology & Microbiology, National Taiwan University, Taipei, Taiwan; 2 Department of Infectious, Parasitic and Immunomediated Diseases, Rome, Italy; 3 Department of Microbiology, University of Tennessee, Knoxville, TN, United States of America; King’s College London Dental Institute, UNITED KINGDOM

## Abstract

ScOpi1p is a well-characterized transcriptional repressor and master regulator of inositol and phospholipid biosynthetic genes in the baker’s yeast *Saccharomyces cerevisiae*. An ortholog has been shown to perform a similar function in the pathogenic fungus *Candida glabrata*, but with the distinction that CgOpi1p is essential for growth in this organism. However, in the more distantly related yeast *Yarrowia lipolytica*, the *OPI1* homolog was not found to regulate inositol biosynthesis, but alkane oxidation. In *Candida albicans*, the most common cause of human candidiasis, its Opi1p homolog, CaOpi1p, has been shown to complement a *S. cerevisiae opi1∆* mutant for inositol biosynthesis regulation when heterologously expressed, suggesting it might serve a similar role in this pathogen. This was tested in the pathogen directly in this report by disrupting the *OPI1* homolog and examining its phenotypes. It was discovered that the *OPI1* homolog does not regulate *INO1* expression in *C. albicans*, but it does control *SAP2* expression in response to bovine serum albumin containing media. Meanwhile, we found that CaOpi1 represses filamentous growth at lower temperatures (30°C) on agar, but not in liquid media. Although, the mutant does not affect virulence in a mouse model of systemic infection, it does affect virulence in a rat model of vaginitis. This may be because Opi1p regulates expression of the *SAP2* protease, which is required for rat vaginal infections.

## Introduction

*Candida albicans* is a commensal organism that lives as a benign resident of the microflora of the human oral, gastrointestinal, and vaginal tracts as well as the skin. It can shift from a commensal to a pathogenic state in response to environmental stimuli that trigger developmental programs that induce the expression of virulence factors. Virulence factors exhibited by *C. albicans* include growth at 37°C, dimorphism, and production of secreted hydrolases such as proteases, lipases, and phospholipases [1, 2].

The pathways that regulate the transcription of secreted aspartyl protease (SAP) virulence factors in *C. albicans* are beginning to be understood, but much remains to be learned. SAPs are encoded by a family of 10 related genes (*SAP1* to *SAP10*) [3, 4]. Unlike *SAP1* to *SAP8*, which encode secreted proteases, *SAP9* and *SAP10* encode GPI-anchored proteases, located at the cell membrane or cell wall, and both are required for virulence [5]. Among this family of genes, Sap2p is the most well-studied protease since it is the major secreted protease during *in vitro* growth conditions. *SAP2* is expressed in *in vitro* conditions where bovine serum albumin is the main nitrogen source [3, 4], and its regulation in these conditions has been well characterized. *SAP2* is under the control of the *STP1* transcription factor and *STP1*’s upstream GATA transcription factors *GLN3* and *GAT1* [6, 7].

The importance of *SAP2* in pathogenesis has been discussed by several groups. For instance, De Bernardis et al. demonstrated that *SAP2* is a major virulence contributor in the rat vaginitis model [8, 9]. Schaller et al. showed that *SAP2* is required to cause tissue damage in an *in vitro* model of vaginal candidiasis [10]. In addition, Hube et al. demonstrated that *SAP2* was required for virulence in a rodent model of systemic infection [11]. In contrast, Naglik et al. and Lermann and Morschhäuser found that *SAP2* was not required to invade and damage oral or vaginal reconstituted human epithelium [12, 13]. Meanwhile, the effect of the aspartic protease inhibitor pepstatin A on reducing tissue damage caused by *C. albicans* in the reconstituted human epithelium model remains elusive. Naglik et al. showed that pepstatin A can attenuate tissue damage, while Lermann and Morschhäuser demonstrated no effect, leaving the role for the Sap family in inducing epithelial damage controversial [12, 13]. Thus, there is contradictory evidence about the role of *SAP2* amd other SAPs in pathogenesis.

*S. cerevisiae OPI1* (*ScOPI1*) is a negative regulator of inositol biosynthesis, and acts to inhibit the transcription of *ScINO1* along with other phospholipid biosynthetic genes in response to extracellular inositol levels [14–17]. *ScINO1* encodes the inositol-3-phosphate synthase (ScIno1p) that catalyzes the conversion of glucose-6-phosphate to inositol-3-phosphate, which is then dephosphorylated by *INM1* or *INM2* to form inositol [17–19]. Inositol and cytidyldiphosphate-diacylglycerol (CDP-DAG) are precursors for the essential phospholipid phosphatidylinositol (PI). ScOpi1p acts as the master regulator of *ScINO1* and other target genes by inhibiting the transcriptional activators ScIno2p and ScIno4p. The mechanism by which it regulates these genes in response to extracellular inositol has been well described [15–17, 20–22].

The structural gene encoding *ScINO1* is conserved between *S. cerevisiae* and *C. albicans*, and shares similar function [23]. *C. albicans* and *S. cerevisiae INO1* homologs are similarly regulated in response to exogenously provided inositol [22]. The *ScOPI1* ortholog in *C. albicans* (*OPI1*) can complement *Scopi1*Δ for *INO1* regulation in *S. cerevisiae* [24]. We therefore hypothesized that *OPI1* might function as an *INO1* negative regulator in *C. albicans* as it does in *Candida glabrata* [25]. However, a report regarding the ScIno2p and ScIno4p homologs suggested that the regulation of *INO1* expression in *S. cerevisiae* and *C. albicans* might not be conserved [26]. The *C. albicans* heterodimeric transcription factors *INO2* and *INO4* (related to *ScINO2* and *ScINO4* from *S. cerevisiae*) did not regulate *INO1*, but instead activated ribosomal protein genes such as *RPL32*. These results indicate that inositol regulation might be transcriptional rewired between these two related eukaryotic organisms. A previous report comparing *S. cerevisiae* and *C. albicans* Gal4p transcription factor homologs that control sugar metabolism suggests that these proteins have been rewired between these two organisms [27]. In *C. albicans* the Gal4p homolog activates the gluconeogenesis gene *LAT1* instead of galactose metabolism genes such as *GAL10*, and surprisingly *GAL10* was activated by another transcription factor, *CPH1*. Therefore, we wished to investigate if *C. albicans OPI1* has a similar role in inositol regulation to *ScOPI1* and *CgOPI1*, or if it has possibly been transcriptional rewired.

In this communication, we report that *C. albicans OPI1* does not regulate the inositol biosynthetic gene *INO1*, but affects the *SAP2* expression and virulence of *C. albicans* in a rat vaginitis model. In addition, *OPI1* affects morphogenesis at 30°C. These results illustrate that the regulation of inositol biosynthesis in *C. albicans* and *S. cerevisiae* is different. From now on, in this paper, all genes from *C. albicans* will be referred to by their simple names such as *OPI1* or *INO1*, whereas genes from other organisms such as *S. cerevisiae* will be referred to as *ScOPI1* or *ScINO1*.

## Materials and Methods

### Ethics Statement

Mouse model of systemic infection studies were conducted in the animal facility at University of Tennessee (UT) in good practice as defined by the United States Animal Welfare Act and in full compliance with the guidelines of the UT Institutional Animal Care and Use Committee (IACUC). The mouse experiments were reviewed and approved by the UT IACUC under protocol number L016. Procedures involving rats and their care were conducted in conformity with national and international laws and policies. The study has been approved by the Committees on the Ethics of Animal Experiments of the Istituto Superiore di Sanita’, Rome, Italy (Permit Number: DM 227/2009-B). All experimental procedures were carried out according to the ARRIVE (Animal Research: Reporting *In Vivo* Experiments; http://www.nc3rs.org.uk/page.asp?id=1357) and NIH (National Institutes of Health) guidelines for the ethical treatment of animals.

### Strains and growth media

*C. albicans* strains used in this study are shown in Table 1. Media used in this study include YPD (yeast extract-peptone-dextrose: 1% yeast extract, 2% peptone, 2% glucose), defined medium 199 (M199, Invitrogen, pH7.0 adjusted by 150mM HEPES), Spider (1% nutrient broth, 1% mannitol, 0.2% dipotassium phosphate, 1.35% agar), YPD containing 10% fetal bovine serum, YCB-BSA (1.17% yeast carbon base-Difco, 0.2% bovine serum albumin-Sigma),YCB-BSA-YE (1.17% yeast carbon base, 0.2% bovine serum albumin, 0.1% yeast extract, pH 5.0) [28, 29]. Unless otherwise stated, agar plates were solidified with 2% agar (granulated, Fisher).

**Table 1:** *Candida albicans* strains used in this study.

**Strains**	**Genotype**	**Parent**	**Source**
SC5314 (wild-type)	Prototrophic wild type	Clinical isolate	[47]
YLC58	*opi1*Δ::*NAT1-FLP*/*OPI1*	SC5314	This study
YLC85	*opi1*Δ/*OPI1*	YLC58	This study
YLC86	*opi1*Δ/*opi1*Δ::*NAT1-FLP*	YLC85	This study
YLC88	*opi1*Δ*/opi1*Δ	YLC86	This study
YLC117	*opi1*Δ*/opi1*Δ::*OPI1-NAT1*	YLC88	This study
SAP2MS4A	*sap2*Δ*/sap2*Δ	SC5314	[37]
YLC223	*OPI1/OPI1 URA3*::*P_ACT1_ -SAP2*	SC5314	This study
YLC226	*opi1*Δ*/opi1*Δ *URA3*::*P_ACT1_ -SAP2*	YLC88	This study

### Strain construction

**Figure 1:**
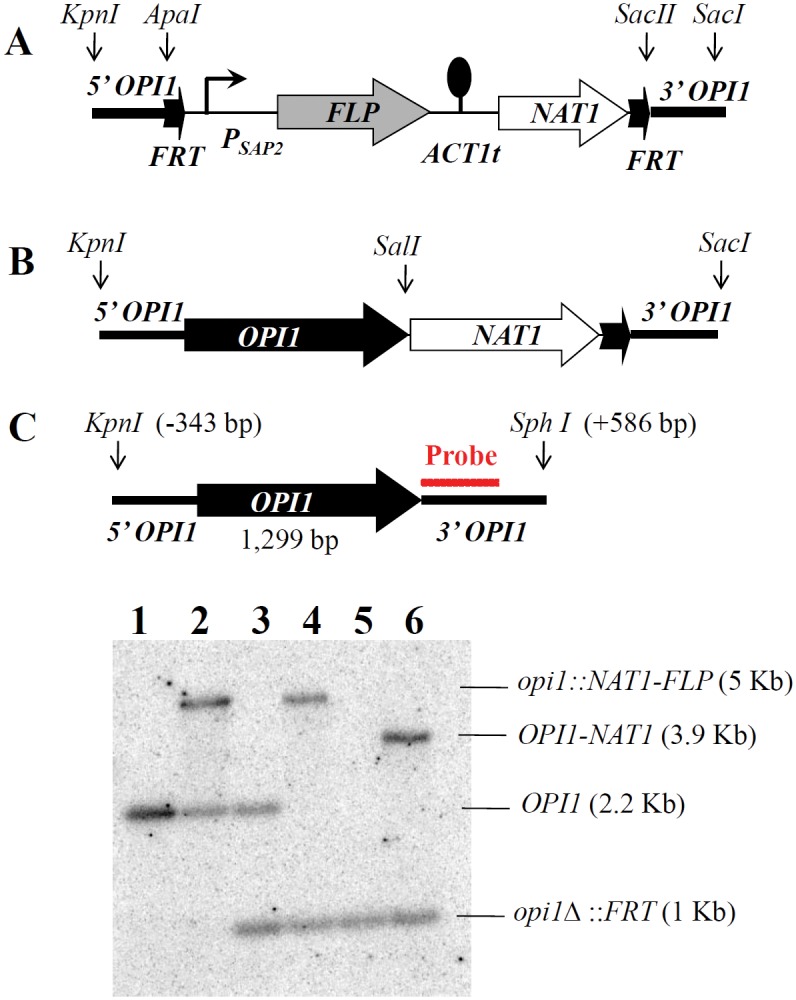
The *OPI1* gene was disrupted in the strain SC5314 using the *CaNAT1*-*FLP* cassette. (A) Structure of the *opi1::CaNAT1-FLP* disruption construct. Approximately 500 base pairs of non-coding DNA flanking the 5’ and 3’ ends of the *OPI1* gene (5’-and 3’-NCR, respectively) were cloned onto either flank of the *CaNAT1-FLP* cassette. The thick dark arrows represent the FRT sites of the *FLP* recombinase. The ball-and-stick symbol represents the *ACT1* terminator (*ACT1*t), and the thinner bent arrow represents the *SAP2* promoter (*P_SAP2_*). (B) The *OPI1-NAT1* construct used to reintegrate *OPI1* into the *opi1*Δ*/*Δ mutant. (C) Southern blotting was used to confirm the *opi1*Δ*/*Δ disruptions. The genomic DNA of the wild type and mutants was cut by *KpnI* and *SphI* restriction enzymes. A PCR product containing the ~500 bp 3’ NCR of *OPI1* (amplified with primers TRO524 and TRO525) was used as a probe (red line) for Southern blot confirmation. Lanes: 1, wild type; 2, *opi1*Δ::*NAT1-FLP*/*OPI1* strain; 3, *opi1*Δ/*OPI1* strain; 4, *opi1*Δ/Δ::*NAT1-FLP* strain; 5, *opi1*Δ/Δ strain; and 6, *opi1*Δ/Δ::*OPI1-NAT1* strain.

The *C. albicans OPI1* gene (*OPI1*) was disrupted by using the *CaNAT1-FLP* cassette [30] (Table 2). For the *OPI1* disruption construct, the 379 base pair (bp) 5’ non-coding region (NCR) of *OPI1* was amplified with primers TRO522 and TRO526 (Table 3), and cloned as a *Kpn*I-*Apa*I digested 228bp fragment into pJK863 5’ of the *CaNAT1-FLP* cassette (Fig. 1A). The 448 bp 3’ NCR of *OPI1* was amplified with primers TRO524 and TRO525 which introduced *Sac*II and *Sac*I sites, and was cloned into pJK863 3’ of the *CaNAT1-FLP*cassette (Fig. 1A). This created the *OPI1* knock out construct plasmid pYLC36 (Table 2, Fig. 1A), which was cut with *Kpn*I and *Sac*I to release the disruption construct (5’ NCR of *OPI1-CaNAT1-FLP*-3’ NCR of *OPI1*) which was transformed into the wild type SC5314 strain by electroporation [31]. The disruption construct was used to sequentially disrupt both alleles of *OPI1*. The *OPI1* reconstitution construct was made by amplifying a 1.7 Kb fragment containing the *OPI1* ORF and 5’ NCR from SC5314 genomic DNA using primers (JCO12 and JCO14) that introduced *Kpn*I and *Sal*I sites. This fragment was ligated into the pRS316 vector along with another 1.7 Kb fragment containing the *NAT1–*3’NCR of *OPI1* amplified from plasmid pYLC36 using primers JCO50 and TRO42 which introduced *Sal*I and *Sac*I sites. This resulted in the *OPI1* reconstitution plasmid pYLC37 (Table 2, Fig. 1B). The 3.4 Kb *Kpn*I-*Sac*I fragment from pYLC37 was transformed into the *opi1*Δ/Δ mutant (YLC88) in order to create the reconstituted *opi11*Δ/Δ::*OPI1* strain (YLC117). The *SAP2* constitutive expression construct was made by cloning the dominant selectable marker *NAT1* with primers JCO129 and JCO130 to *Nde*I-digested pAU34 [32] and resulted in pYLC219. The *SAP2* ORF was then cloned to *Xma*I-digested pYLC219 with primers JCO131 and JCO132, and resulted in pYLC221, which can constitutively express *SAP2* under the control of the *ACT1* promoter. To transform this constitutive construct into wild type and *opi1*Δ/Δ strains, a *Ppu*MI-digested linear plasmid pYLC221 was integrated at the *URA3* site of *Candida* genome, and resulted in *OPI1*/*OPI1 URA3*::*P_ACT1_-SAP2* (YLC223) and *opi1*Δ/Δ *URA3*::*P_ACT1_-SAP2* (YLC226).

**Table 2:** Plasmids used in this study.

**Plasmids**	**Characteristics**	**Source**
pJK863	*CaNAT1-FLP* cassette carrying nourseothricin resistance gene	[30]
pYLC36	pJK863 flanked 5’ and 3’*OPI1* ^NCR^ for *OPI1* gene knock out	This study
pYLC37	*OPI1* reconstitution construct	This study
pAU34	Constitutively expression construct under the control of *ACT1* promoter	[32]
pYLC219	pAU34 contained *NAT1* selectable marker	This study
pYLC221	pYLC219 contained *SAP2* ORF for constitutive expression	This study

**Table 3:** Primers used in this study.

**Primer**	**Use**	**Sequence (5’ à 3’)**
JCO522	Disrupt *OPI1*	AAAAAAGGGCCCTACACACACACACACTTACACACAT
JCO526	Disrupt *OPI1*	AAAAAAGGGCCCCGGTTTCCCCCTTTTTATATA
JCO524	Disrupt *OPI1*	AAAAAACCGCGGTGAGTGGTGGTTTCTTTTTGTT
JCO525	Disrupt *OPI1*	AAAAAAGAGCTCCAAGTTGGACTACAAATGGTCAAG
JCO12	Restore *OPI1*	GTGTGGTACCTATTTCCAAATTCA
JCO14	Restore *OPI1*	AAAAGTCGACCTACTACACACTACCATCTA
TRO369a	Confirm *OPI1* disruptions	GCACGTCAAGACTGTCAAGG
TRO532	Confirm *OPI1* disruptions	ACGGCATGCTAGGTATATACTGCT
JCO48	*ACT1* Northern blot probe	CCAGCTTTCTACGTTTCC
JCO49	*ACT1* Northern blot probe	CTGTAACCACGTTCAGAC
JCO35	*SAP2* Northern blot probe	TTCATTGCTCTTGCTATTGCT
JCO36	*SAP2* Northern blot probe	CACCGGCTTCATTGGTTTTA
TRO562	*INO1* Northern blot probe	GAAAACTCTGTTGTTGAAAAAGATG
TRO563	*INO1*Northern blot probe	TTGTTGGCACGTTCACTTTG
JCO129	*SAP2* constitutive expression	AAAAAACATATGCCCCGCGGGATATCAAGC
JCO130	*SAP2*constitutive expression	AAAAAACATATGTGGGTACCGAATTCGAGCT
JCO131	*SAP2* constitutive expression	AAAAAACCCGGGATGTTTTTAAAGAATATTTTC
JCO132	*SAP2* constitutive expression	AAAAAACCCGGGTTAGGTCAAGGCAGAAATAC
JCO133	*SAP2*constitutive expression	AGAGAGCAGAAACTCATGCCT
JCO134	*SAP2* constitutive expression	TAAGCATTCCAACCAGCATC
JCO122	*SAP2*constitutive expression	AAAAAAGGTACCCGTCAAAACTAGAGAATAATAAAG
JC798	*SAP2* Real time PCR primer	TCCTGATGTTAATGTTGATTGTCAAG
JC799	*SAP2* Real time PCR primer	TGGATCATATGTCCCCTTTTGTT

### Northern blot analysis

Northern blotting for *SAP2* and *INO1* expression was performed as described [33, 34] with the following exceptions. Strains grown in YCB-BSA or YCB-BSA-YE medium at 37°C for 12 hrs (for *SAP2*) and in liquid medium 199 (pH 7.0) at 37°C for 2 hrs (for *INO1*) were collected for total RNA extraction by the hot phenol method. The PCR product containing bps 17–571 of the *SAP2* ORF (primers JCO35 and JCO36) and bps 76–581 of the *INO1* ORF (primers TRO562 and TRO563) were used as probes. Expression was normalized against *C. albicans ACT1* gene expression probed on the same membrane. The *ACT1* probe was generated with the primers JCO48 and JCO49.

### RT real-time PCR

Strains were cultured overnight in YPD at 37°C, washed twice with dH_2_O, then diluted to 0.2 O.D_600_/ml and incubated in liquid YCB-BSA medium (1.17% yeast carbon base, 0.2% BSA) for 12 hrs at 37°C with shaking at 200 rpm. The 50 ml cultures were pelleted at 3000rpm at 4°C and immediately frozen with liquid nitrogen to stop cellular processes. Total RNAs were extracted with a RiboPure Yeast RNA Purification Kit (Ambion), treated with TURBO DNA-*free* Kit (Invitrogen), and 2 μg of DNA-free total RNAs was reverse transcribed to cDNA using High-Capacity cDNA Reverse Transcription Kit (Applied Biosystems). Real-time PCR reactions of 20 μl included 6 ng cDNA (in 6 μl), 10 μl of 2x qPCR master mix (Fast SYBR Green Master Mix; Applied Biosystems), 2 μl of 2.5 μM forward primer (JC798 for *SAP2*), 2 μl of 2.5 μM reverse primer (JC799 for *SAP2*). Primer design for detecting *SAPs* expression was based on previous publication by Naglik et al [13]. Quantitative PCR conditions were shown below 95°C /10 min for denaturation; 95°C /3 sec, 60°C /30 sec (40 cycles); 95°C /15 sec, 60°C /60 sec, 95 °C /15 sec (melting curve). The StepOnePlus System and StepOne v2.2 (Applied Biosystems) were used to determine ∆∆Ct. The bar graphs of *ACT1* normalized relative quantity compared with wild-type (SC5314) were created with Prism 5.03.

### Southern blot analysis

Hybridization conditions for the Southern blot analysis were similar to those for Northern blot analysis, except that the Techne Hybrigene oven was set to 60°C for the incubation step, and 42°C and 60°C for washing steps. The cells were grown in liquid YPD at 30°C overnight. The genomic DNA was extracted using the Winston-Hoffman method [35] and 20μg of genomic DNA were subjected to Southern blotting. The genomic DNA of the wild type and *opi1*Δ/Δmutants was cut by *Kpn*I and *Sph*I restriction enzymes. PCR products containing the ~500bp 3’ NCR of *OPI1*(primers TRO524 and TRO525) were used as probes for Southern blot confirmation (Fig. 1C).

### Mouse bloodstream infection studies

Five- to six-week-old male CD1 mice (18 to 20 g) from Charles River Laboratories were used in this study. Mice were housed at five per cage. For infection, colonies from each *C. albicans* strain were inoculated into 20 ml of YPD. Cultures were grown overnight at 30°C with shaking in YPD, washed twice with 25 ml of sterile water, counted by hemocytometer, and resuspended at 10^7^ cells per ml in sterile water. Mice were injected via the tail vein with 0.1 ml of the cell suspension (10^6^ cells), and the course of infection was monitored for up to 14 days. The survival of mice was monitored twice daily, and moribund mice (body weight reduced by 30%, unable to eat/drink, or severely hunched) were euthanized with CO_2_. Cells were also plated on YPD to determine the viability. At least two independent infections were performed for each strain. The statistical analysis was done using Prism 5.03 software (GraphPad Software). For the mouse model of systemic infection, Kaplan-Meier survival curves were compared for significance using the Mantel-Haenszel log rank test. Statistical significance was set at *P*< 0.05.

### Rat vaginitis studies

The protocol of estrogen-dependent rat vaginal infection model adapted from De Bernardis et al. [8] was used throughout this study. Briefly, oophorectomized female Wistar rats (80–100 g; Charles River, Calco, Italy) were injected subcutaneously with 0.5 mg of estradiol benzoate (Benzatrone; Samil, Rome). Six days after the first estradiol treatment, the animals were inoculated intravaginally with 10^7^ yeast cells of each *C. albicans* strain in 0.1 mL. The inoculum was dispensed into the vaginal cavity through a syringe equipped with a multipurpose calibrated tip (Combitip; PBI, Milan, Italy). The yeast cells had been previously grown in YPD broth at 28°C on a gyratory shaker (200 rpm), harvested by centrifugation (1500 g), washed, counted in a hemocytometer, and suspended to the required number in saline solution. The results of two independent experiments are each represented separately. A third experiment involving all of the strains is not shown, but gave similar trends. In each experiment, each *Candida* strain was inoculated into 5 rats. Kinetics of *C. albicans* growth in, and clearance from, the vaginal cavity was measured by colony forming unit (CFU) enumeration after culturing 100 μl of vaginal samples, taken by washing the vaginal cavity by gentle aspiration of 100 μl of sterile saline solution, repeated four times, at 1:10 serial dilutions on Sabouraud agar containing chloramphenicol (50 μg/ml). CFUs were enumerated after incubation at 28°C for 48 h.

## Results

### *C. albicans OPI1* does not regulate *INO1* expression

When heterologously expressed in an *S. cerevisiae Scopi1∆* mutant, the *C. albicans OPI1* gene has been demonstrated to repress expression of a reporter gene that contains the inositol/choline responsive element (ICRE) found in *ScINO1* and other ScOpi1p-ScIno2p-ScIno4p target genes [24]. This data suggested that *C. albicans* Opi1p may regulate the cognate *C. albicans INO1* gene, as its homolog does in *S. cerevisiae*. In order to test this both copies of the *C. albicans OPI1* gene were disrupted in *C. albicans* using the *CaNAT1-FLP* cassette [30] as described in Fig. 1.

**Figure 2:**
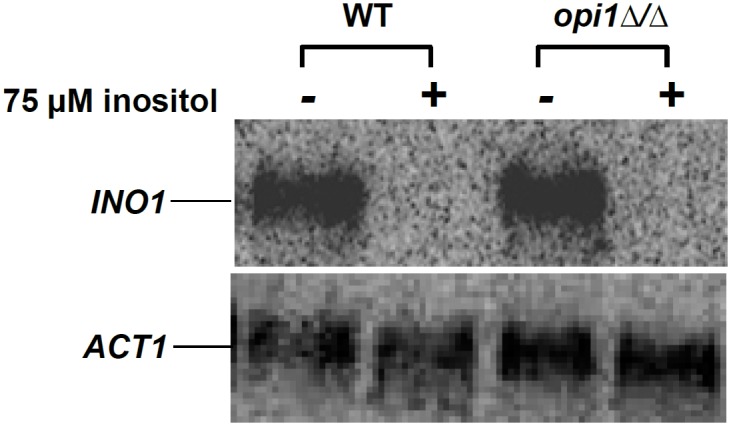
*C. albicans OPI1* does not regulate *INO1* expression. Strains were grown for 2 hrs in Medium 199, pH7.0 (± 75 μM inositol) at 37°C, collected, and subjected to Northern blotting against *INO1*. *ACT1* was reprobed on the same membrane as a loading control.

The wild type and *opi1*Δ/Δ strains were then compared to see if the *opi1∆/∆* mutant would fail to repress *INO1*, as expected, if it acts like the homologous *S. cerevisiae* mutant, *Scopi1∆* [22]. First, the strains were grown in Medium 199, pH 7.0, which contains low levels of inositol (~10 μM), which should result in high expression of *INO1*, and it was found that both upregulated *INO1* to similar levels (Fig. 2). Then, they were grown in the same medium supplemented with 75 μM inositol, which should repress *INO1* expression in wild-type, but not in the *opi1∆/∆* mutant, if it cannot repress the gene. However, in both strains, *INO1* was similarly repressed, suggesting that inositol biosynthesis is regulated by different transcription factors in *C. albicans*.

### The *opi1*Δ/Δ mutant exhibits hyperfilamentous growth in filament-inducing media at 30°C

**Figure 3:**
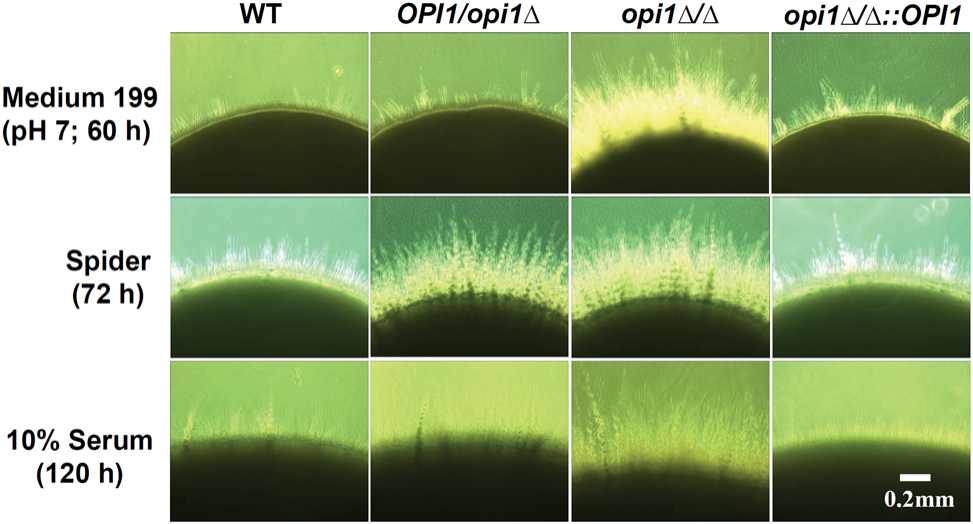
The *opi1*∆/∆ mutant exhibits hyperfilamentous growth at 30°C in filament-inducing conditions on agar plates. *C. albicans* strains from overnight cultures were diluted in sterile water and plated on the indicated medium and grown at 30°C for the indicated amount of time.

It has been shown that *ScOPI1* is necessary to activate invasive growth and *ScFLO11* expression in *S. cerevisiae* [34]. It was therefore hypothesized that *OPI1* would affect filamentous growth in *C. albicans*. Three filament-inducing media were used to test this hypothesis. In contrast to the situation with the *Scopi1∆* mutant in *S. cerevisiae*, it was found that the *opi1*Δ/Δ mutant exhibited hyperfilamentous growth rather than hypofilamentous growth, but only at 30°C on solid filament-inducing agar plates (Fig. 3). This effect was not observed at 37°C on similar media. These phenotypes were also not seen in liquid forms of the same filament-inducing media at either 30°C or 37°C. In order to control for a possible effect from some other unlinked mutation, a copy of the *C. albicans OPI1* gene was reintegrated into the *opi1∆/∆* mutant (Fig. 1B), and it was found that the phenotype was restored when the wild-type copy of *OPI1* was present (Fig. 3), indicating that the hyperfilamentous growth at 30°C is linked to the loss of *OPI1* gene.

### *OPI1* does not affect virulence in a mouse model of systemic infection

**Figure 4:**
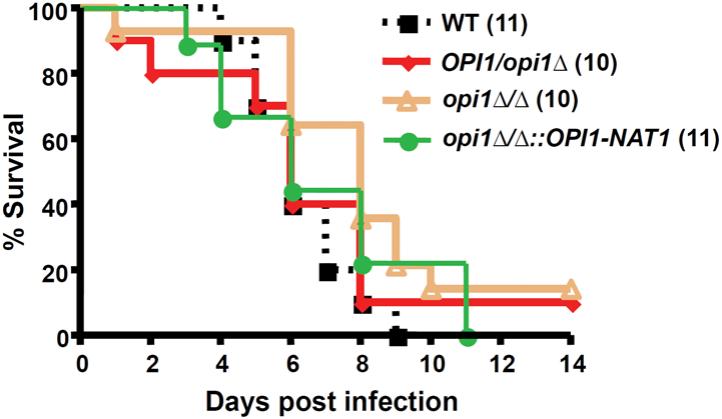
*OPI1* is not required for virulence of *C. albicans* in a mouse model of systemic candidiasis. Each strain was used to infect mice by injecting 10^6^ *C. albicans* yeast-form cells into the tail-vein of each mouse. The mice were then assessed over the course of 14 days. The number of mice used for a specific strain is indicated in parentheses. The data obtained here are from a single experiment.

The *opi1∆/∆* mutant appears to affect the ability of the fungus to repress filamentation at lower temperatures. Some hyperfilamentous mutants such as *nrg1∆/∆* and *tup1∆/∆* have been found to be attenuated in virulence in mouse models of infection [36]. Therefore, a mouse model of systemic infection was used to test the role of *OPI1* in virulence. However, the *OPI1* gene does not contribute to the virulence in this model since the *opi1*Δ/Δ mutant exhibits a similar phenotype to wild-type on the survival curves of mice (Fig. 4).

### *OPI1* is involved in establishing infection in the rat vaginitis model

**Figure 5:**
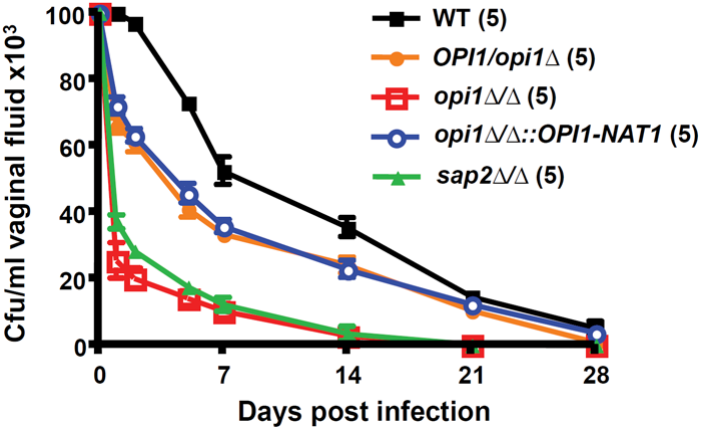
*OPI1* is involved in establishing infection in the rat vaginitis model. For each *C. albicans* strain, 5 rats were inoculated on day 0 with 10^7^ blastospores, and vaginal colony forming units (CFUs) at particular time points were counted by plating at the indicated time points. The error bars represented the standard errors of the mean in each group. The number of rats used for a specific strain is indicated in parentheses and the data obtained here are from a single experiment.

In addition to infections of the bloodstream, *C. albicans* can also cause infections of mucosal surfaces including the vaginal tract [8]. A rat vaginitis model was used to determine if the *opi1∆/∆* mutation would play a role in the establishment of infection in this host niche. It was demonstrated that *OPI1* was involved in establishing rat vaginitis. In this model *C. albicans* cells are injected into the rat vaginal tract, and then over time the level of colonization is measured based on the recovery of colony counts. It was discovered that the *opi1*Δ/Δ mutant is quickly cleared by the host compared to the wild type (Fig. 5). The *opi1∆/∆::OPI1* reintegrant strain and *opi1∆/OPI1* heterozygous mutant had an intermediate phenotype between the *opi1*Δ/Δ mutant and wild type (Fig. 5).

It has been shown that deletion of the *C. albicans SAP2* proteasegene [8] causes a similar clearance to the *opi1∆/∆* mutant, and a *sap2∆/∆* mutant (SAP2MS4A) [37] was included in this experiment as a control. Our results confirmed that an independently constructed *sap2∆/∆* mutant (gift from Joachim Morschhäuser), behaved like a previously constructed *sap2∆/∆* mutant, and exhibits reduced colonization in the rat vaginal tract (Fig. 5), suggesting the importance of *SAP2* in the rat vaginitis model. Our results also indicate that *OPI1* plays a critical role in establishing infection in the rat vaginal tract (Fig. 5).

### *OPI1* affects rat vaginal establishment through regulating *SAP2*

**Figure 6:**
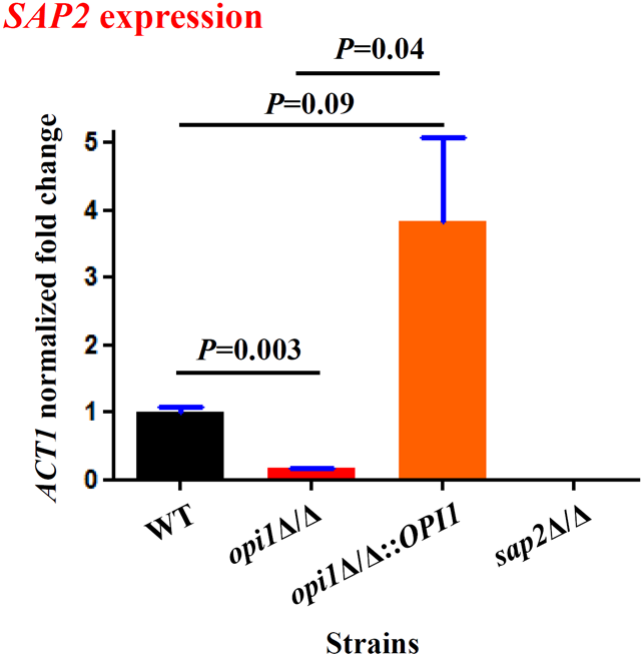
*SAP2* expression is regulated by *OPI1* in *C. albicans*. RT real-time PCR was used to assess the *SAP2* expression levels in the wild-type (SC5314), *opi1*Δ/Δ, *opi1*Δ/Δ::*OPI1* and *sap2* Δ/Δ mutants. Strains were cultured overnight in YPD at 37°C, washed twice with dH_2_O. Then strains were diluted to 0.2 O.D_600_/ml and incubated in liquid YCB-BSA medium (1.17% yeast carbon base, 0.2% BSA) for 12 hrs at 37°C. The error bars represented the standard errors of the mean. The data obtained here are from a representative single experiment with technical triplicates. *P* value was determined by *t* tests and < 0.05 was considered statistically significance.

The similarity of the phenotypes of the *opi1∆/∆* mutant with the *sap2∆/∆* mutant suggested that *OPI1* might act through *SAP2*. In wild-type cells, *SAP2* is upregulated in bovine serum albumin (BSA) media. We performed reverse transcriptase (RT) real-time PCR to detect if *OPI1* controls *SAP2* expression in YCB-BSA medium. The *opi1∆/∆* mutant showed 5.5 fold reduced *SAP2* expression compared to wild type (Fig. 6), indicating that *OPI1* controls *SAP2* expression. The *opi1∆/∆*::*OPI1* reintegrant strain can restore the *SAP2* expression and actually shows ~ 3 fold higher expression of *SAP2* than the wild type.

**Figure 7:**
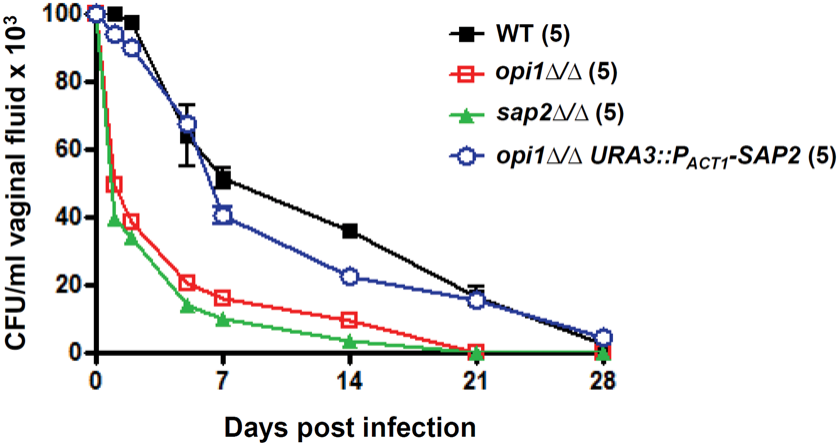
*OPI1* affects rat vaginal establishment through regulating *SAP2*. For each strain 5 rats were inoculated on day 0 with 10^7^ blastospores, and vaginal CFUs were counted by plating at the indicated time points. The error bars represented the standard errors of the mean in each group. The number of rats used for a specific strain is indicated in parentheses and the data obtained here are from a single experiment.

In order to test if *OPI1* affects colonization of the rat vaginal tract through *SAP2*, an epistasis experiment was performed in which the *SAP2* gene was overexpressed in the *opi1∆/∆* mutant via the *ACT1* promoter (*P_ACT1_-SAP2*). This overexpression was confirmed by Northern blotting (S1 Fig.). If *opi1∆/∆* blocked rat colonization by compromising *SAP2* expression, then overexpression of *SAP2* from an independent promoter should suppress the phenotype. In contrast to the *opi1∆/∆* mutant, the *opi1*Δ/Δ*URA3::P_ACT1_-SAP2* mutant was suppressed for its defect in rat vaginal infection, and behaved similarly to the wild type (Fig. 7). This implicates the *OPI1* gene as a regulator of *SAP2* in the vaginal tract of the rat.

## Discussion

Our results show that the *OPI1* gene of *C. albicans*, unlike its homologs in *S. cerevisiae* and *C. glabrata* [22, 25], does not affect *INO1* expression, but does repress filamentous growth at low temperature (Fig. 3) and regulates virulence in the rat vaginitis model (Figs. 5 and 7). The latter phenotype appears to be mediated by changes in *SAP2* expression. The *opi1*Δ/Δ mutant exhibits reduced *SAP2* expression compared with the wild type in liquid YCB-BSA medium (Fig. 6), and *in vivo SAP2* overexpression can restore the *opi1*Δ/Δ mutant’s vaginal colonization defect, when under the control of the constitutive *ACT1* promoter. Epistasis experiments are inherently challenging to interpret, as overexpression of a target gene such as *SAP2* could lead to enhanced colonization by a mechanism that bypasses the actual defect caused by the *opi1*∆/∆ mutation. Based on our data, this possibility cannot be completely ruled out. It is also possible that *OPI1* controls colonization in the rat vaginal tract by regulating one of the other *SAPs* (e.g. *SAP1*, *SAP3–10*). However, as the differential expression of nine other *SAPs* in the *opi1∆/∆* mutant compared to the wild type using the same condition (i.e. YCB-BSA liquid medium and RT real time PCR) was not detected, we do not know if these others are affected *in vivo*, and this remains to be examined (unpublished data). Meanwhile, further studies will be needed to test if *SAP4*, *SAP5* and *SAP6* genes are regulated by *OPI1* under hypha-inducing conditions since *SAP4–6* are hypha-specific genes [38, 39].

In *S. cerevisiae*, ScOpi1p is the master regulator of *ScINO1* and other phospholipid genes [15–17, 20]. ScOpi1p controls expression in response to cellular inositol levels by binding to Ino2p in the Ino2p-Ino4p heterodimer and repressing its activation of *ScINO1*, among other targets. When inositol is plentiful, PI is efficiently synthesized from CDP-DAG and inositol by the ScPis1p enzyme [40, 41]. In this circumstance, the endoplasmic reticulum (ER) localized pool of phosphatidic acid (PA), which is the precursor for CDP-DAG, is consumed, and ScOpi1p is translocated to the nucleus. There, it binds ScIno2p and represses *ScINO1* with help from the global repressor Sin3p via a direct interaction involving the N-terminal Sin3p binding domain of ScOpi1p [16, 24]. When inositol is not plentiful in the environment, cellular stores drop and PI synthesis slows causing a build-up of precursors including PA. ScOpi1p binds to PA in the ER via its basic domain, and the ER membrane protein Scs2p via its FFAT domain [20, 42]. This sequesters ScOpi1p to the ER, and then Ino2p-Ino4p activate transcription of *ScINO1* so inositol can be synthesized for PI production.

A previous report demonstrated that *OPI1* from *C. albicans* could complement an *Scopi1∆* mutant in *S. cerevisiae*, and it could repress the ICRE promoter element found on *ScINO1* and other phospholipid biosynthetic genes when expressed heterologously in *S. cerevisiae* [24]. However, we found that in *C. albicans, OPI1* does not regulate *INO1* expression. This overlap in function of *CaOPI1* when expressed heterologously in *S. cerevisiae*, but not endogenously in *C. albicans*, may be due to the conservation of some key domains required for ScOpi1p function, but not the conservation of other domains (S2 Fig.). In particular, the *C. albicans* Opi1p has very little conservation with ScOpi1p in the large N-terminal ScSin3p binding domain [16]. However, CaOpi1p does have some conserved sequences with the C-terminal ScIno2p interaction domain of ScOpi1p, including two out of three residues (ScOpi1p aas 358–360) that were shown to be crucial for ScIno2p-ScOpi1p interactions in *S. cerevisiae* [16]. In contrast, CaOpi1p shares very few residues in common with ScOpi1p in the PA-binding basic domain, and no residues of the FFAT domain that binds to ScScs2p [20, 42]. It does, however, carry a leucine zipper motif with some isoleucine substitutions that has been shown to be crucial for ScIno2p-ScOpi1p interactions [15]. Thus, this conservation of some domains, but not others may help explain why CaOpi1p can complement a *Scopi1∆* mutant for *ScINO1* repression [24], but not act the same within *C. albicans* itself. Further support for our findings comes from the observation that *CaINO2* and *CaINO4* do not appear to regulate *CaINO1* either, but may actually regulate ribosomal genes [26]. This is in marked contrast to *CgOPI1* from *C. glabrata*, which does regulate *CgINO1* with help from *CgINO2* and *CgINO4* [25]. Consistently, CgOpi1p has close conservation of all of the important regulatory domains of ScOpi1p (S2 Fig.). Interestingly, one other ScOpi1p homolog has been characterized, and this is Yas3p from *Yarrowia lipolytica*. Yas3p also does not have a number of domains conserved with ScOpi1p, and like CaOpi1p does not regulate *YlINO1*, but does, along with Ino2p and Ino4p homologs Yas1p and Yas2p, respectively, regulate hexane metabolism genes [43]. The *C. albicans* regulators *of CaINO1* are currently unknown, and this will be interesting to elucidate, as expression of *CaINO1* is regulated by extracellular inositol levels, but not by CaOpi1p and apparently not by CaIno2p or CaIno4p either.

Finally, the role Opi1p in repressing filamentation at 30°C on solid media remains elusive (Fig. 3). The *opi1*Δ/Δ mutant exhibits hyperfilamentous growth in filament-inducing agar plates including medium 199, spider, and 10% serum at 30°C, but not 37°C. These results indicate that *OPI1* might be a low temperature repressor of filamentous growth. It has been demonstrated that *C. albicans CPP1*, a tyrosine phosphatase, is required tor repress the yeast to hyphal transition at 23°C in contact with solid surfaces [44, 45]. The *cpp1*Δ/Δ mutant exhibited hyperfilamentous growth on spider and a wide variety of rich and defined solid media including Lee’s medium, YPD, YPM, and 10% serum at 23°C, but not at 37°C. The germ tube formation defect of the *cpp1*Δ/Δ mutant was not observed at liquid culture at 37°C, an effect similar to *opi1*Δ/Δ mutant. In contrast to *opi1*Δ/Δ, the *cpp1*Δ/Δ mutant exhibited reduced virulence in mouse systemic infection and mouse mastitis models [44–46]. The relationship between Opi1 and Cpp1 is unknown and needs further studies in *C. albicans*. Taken together, our data suggest that, when compared to its homolog in *S. cerevisiae*, *C. albicans* has a transcriptionally rewired regulator, *OPI1*, which does not regulate *INO1* expression but affects morphogenesis, *SAP2* expression and virulence in a rat vaginitis model. It also makes it clear that identification of ScOpi1p homologs in other fungi does not clearly implicate them for roles in regulating inositol biosynthesis in these microbes. Rather, the Opi1p family members, which are conserved in a wide variety of fungi appear to have a diversity of functions.

## Supporting Information

S1 Fig *SAP2* is overexpressed in the *P_ACT1_-SAP2* construct. Expression was tested by Northern blotting in YPD media, and it was confirmed that the *P_ACT1_-SAP2* construct overexpressed *SAP2*.
(TIF)

S2 Fig Multiple sequence alignment comparing Opi1p homologues of *S. cerevisiae* (S.C.), *C. glabrata* (C.G.), *Y. lipolytica* (Y.L.), and *C. albicans* (C.A.). This alignment was performed ****using Clustal W. 2.0.1.0. Asterisk represents conservation among all four species. Various domains represented by different colors, and boxes highlight particularly conserved regions between species. Blue: Opi1-Sin3 interaction domain. Gold: phosphatidic acid (PA)-binding domain. Red: Leucine zipper. Green: FFAT (2 phenylalanines and an acid tract). Purple: Polyglutamine tract. Orange with black boxes: Ino2p activator interaction domain.
(TIF)
