# Supplementary figures and images for: Candida albicans OPI1 Regulates Filamentous Growth and Virulence in Vaginal Infections, but Not Inositol Biosynthesis
Source: PLoS One. 2015 Jan 20;10(1):e0116974. doi: 10.1371/journal.pone.0116974 (PMC4300220; doi:10.1371/journal.pone.0116974)

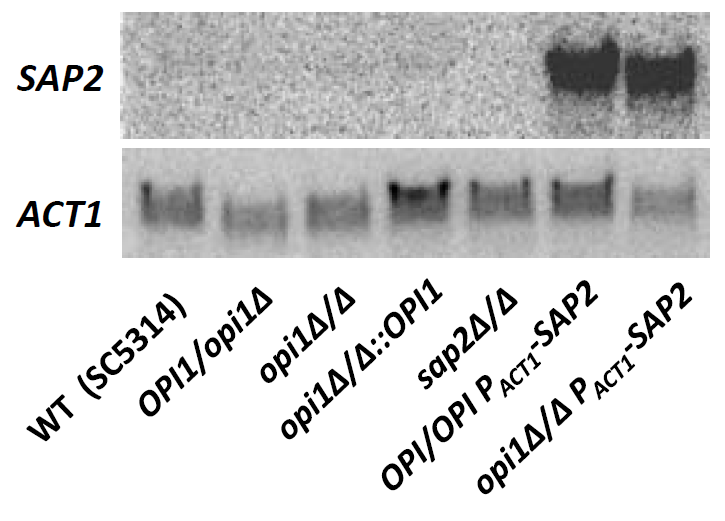

Supplement: S1 Fig — Expression was tested by Northern blotting in YPD media, and it was confirmed that the PACT1-SAP2 construct overexpressed SAP2. (TIF) [file pone.0116974.s001.tif]

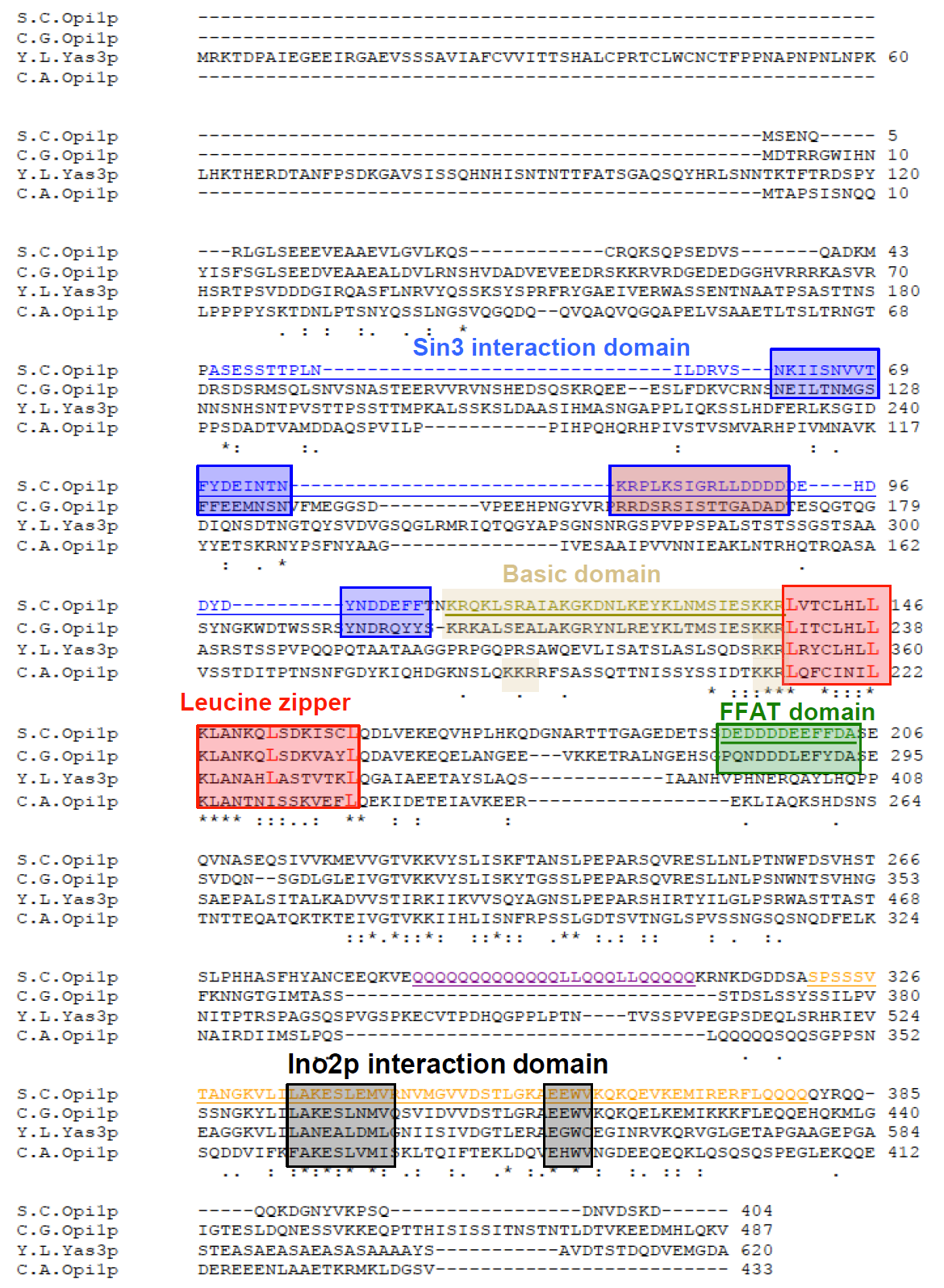

Supplement: S2 Fig — This alignment was performed using Clustal W. 2.0.1.0. Asterisk represents conservation among all four species. Various domains represented by different colors, and boxes highlight particularly conserved regions between species. Blue: Opi1-Sin3 interaction domain. Gold: phosphatidic acid (PA)-binding domain. Red: Leucine zipper. Green: FFAT (2 phenylalanines and an acid tract). Purple: Polyglutamine tract. Orange with black boxes: Ino2p activator interaction domain. (TIF) [file pone.0116974.s002.tif]
